# Survival impact of treatment for chronic obstructive pulmonary disease in patients with advanced non-small-cell lung cancer

**DOI:** 10.1038/s41598-021-03139-5

**Published:** 2021-12-08

**Authors:** Hitomi Ajimizu, Hiroaki Ozasa, Susumu Sato, Tomoko Funazo, Yuichi Sakamori, Takashi Nomizo, Kiyomitsu Kuninaga, Tatsuya Ogimoto, Kazutaka Hosoya, Masatoshi Yamazoe, Takahiro Tsuji, Hironori Yoshida, Ryo Itotani, Kentaro Ueno, Young Hak Kim, Shigeo Muro, Toyohiro Hirai

**Affiliations:** 1 Department of Respiratory Medicine, Kyoto University Graduate School of Medicine, 54 Kawahara-cho, Shogoin, Sakyo-ku, Kyoto, 606-8507 Japan; 2 Department of Respiratory Medicine, Nara Medical University, 840 Shijo-cho, Kashihara, Nara 634-8522 Japan; 3 Department of Biomedical Statistics and Bioinformatics, Graduate School of Medicine, Kyoto University, 54 Kawahara-cho, Shogoin, Sakyo-ku, Kyoto, 606-8507 Japan

**Keywords:** Chronic obstructive pulmonary disease, Lung cancer

## Abstract

Chronic obstructive pulmonary disease (COPD) may coexist with lung cancer, but the impact on prognosis is uncertain. Moreover, it is unclear whether pharmacological treatment for COPD improves the patient’s prognosis. We retrospectively investigated patients with advanced non-small-cell lung cancer (NSCLC) who had received chemotherapy at Kyoto University Hospital. Coexisting COPD was diagnosed by spirometry, and the association between pharmacological treatment for COPD and overall survival (OS) was assessed. Of the 550 patients who underwent chemotherapy for advanced NSCLC between 2007 and 2014, 347 patients who underwent spirometry were analyzed. Coexisting COPD was revealed in 103 patients (COPD group). The median OS was shorter in the COPD group than the non-COPD group (10.6 vs. 16.8 months). Thirty-seven patients had received COPD treatment, and they had a significantly longer median OS than those without treatment (16.7 vs. 8.2 months). Multivariate Cox regression analysis confirmed the positive prognostic impact of COPD treatment. Additional validation analysis revealed similar results in patients treated with immune checkpoint inhibitors (ICIs). Coexisting COPD had a significant association with poor prognosis in advanced NSCLC patients if they did not have pharmacological treatment for COPD. Treatment for coexisting COPD has the potential to salvage the prognosis.

## Introduction

Chronic obstructive pulmonary disease (COPD) is a major public health problem. In 2020, COPD was projected to rank fifth worldwide in terms of disease burden and third in terms of mortality^1,2^. Lung cancer is also one of the leading causes of death in many countries^3^. Several clinical reports have shown that the proportion of deaths from lung cancer in patients with COPD ranges from 4 to 33%^1,4,5^. The frequency of coexisting COPD has been reported to be 40–70% among lung cancer patients^6–9^.

Additionally, airflow limitations have been reported to be a significant risk factor for lung cancer, and share the same risk for the COPD pathogenesis, especially cigarette smoke exposure^5,10–12^. The pathological feature of COPD is the chronic inflammation in pulmonary alveoli caused by the activation of various cytokines by the stimulation of harmful substances within cigarette smoke or other environmental gases^13–15^. Consequently, chronic inflammation alters normal alveolar architecture, promotes emphysema, and stimulates cell proliferation and genetic mutation, affecting the development of lung cancer^15,16^. A hypothesis of a common mechanism of COPD and lung cancer^17^, which has recently attracted considerable attention, was proposed in 2008.

Coexisting COPD may cause severe dyspnea, exacerbation, and poor outcome, and it is possible that patients with lung cancer and coexisting COPD may have a much worse prognosis than those without COPD. In fact, some clinical studies have reported that patients with early-stage non-small-cell lung cancer (NSCLC) with COPD have a poor outcome relative to patients with lung cancer without COPD^18,19^. However, in patients with advanced lung cancer, the impact of coexisting COPD is uncertain. Moreover, it has not been fully examined whether the treatment of COPD might be associated with the prognosis of patients with advanced NSCLC.

Treatment for COPD has advanced in the past two decades due to modifications of older compounds, resulting in more potent, longer-acting drugs that can be delivered via improved devices^20^. Currently, long-acting bronchodilators (LABDs) are the standard treatment for COPD, and these LABDs have been reported to be associated with improvements in lung function, exercise capacity, quality of life, the rate of exacerbations, and prognosis^21–25^.

We hypothesize that treatment intervention for COPD might improve the prognosis of advanced NSCLC patients with COPD. We retrospectively examined the impact of COPD and pharmacological treatment for COPD on the survival outcomes of patients with locally advanced NSCLC in the present study.

## Methods

### Patients

The study flow chart is shown in Supplemental Fig. S1, Additional File 1. We enrolled patients with lung cancer who underwent chemotherapy for recurrence after curative treatment and locally advanced or metastatic NSCLC at Kyoto University Hospital from April 2007 to March 2014.

The entry criteria were as follows: pathological diagnosis of NSCLC, age > 40 years, and performance status (PS) better than 4. Curative treatment was defined as treatment with radiation therapy, radiochemotherapy, or surgery in the present study. The exclusion criteria were as follows: no spirometry, a combination of other respiratory diseases, such as asthma, the use of immunotherapy, including immune checkpoint inhibitors (ICIs), and no smoking history data.

Spirometry was conducted using a Chestac-8800 (Chest M.I., Inc., Tokyo, Japan), and the results obtained met the requirements of the Japanese Respiratory Society guidelines^26^. COPD was diagnosed based on smoking status as previously described^27–30^, and the functional definition, i.e., a forced expiratory volume in 1 s (FEV1) to forced vital capacity (FVC) ratio less than 70%, in accordance with the documents of the Global Initiative for Chronic Obstructive Lung Disease (GOLD)^31^.

### Data collection

The primary endpoint of this study was overall survival (OS), measured from the date of initiation of 1st-line chemotherapy to the date of death (event) or last known date of survival (censored). Data were collected from lung cancer patient records of Kyoto University Graduate School of Medicine. All patients’ vital statuses were confirmed in September 2015.

### Pharmacological treatment of COPD

The use of COPD treatment was also collected from the medical records of our hospital. We defined the use of LABDs and inhaled corticosteroids with/without short-acting bronchodilators as pharmacological treatment of COPD according to GOLD documents^31^. We classified the COPD group into two groups, with COPD treatment and without COPD treatment, depending on the presence or absence of treatment.

### Ethics approval and consent to participate

This study was approved by the Ethics Committee of Kyoto University Graduate School and Faculty of Medicine (http://www.ec.med.kyoto-u.ac.jp) (approval No. R0702-1) and performed in accordance with the Declaration of Helsinki, and informed consent from the patients was waived because this is a retrospective study. We have disclosed information about this study on the official site of our laboratory (http://kukonai.com/).

### Statistical analysis

For comparisons of background features between the non-COPD and COPD groups or between the patients with COPD treatment and without COPD treatment, continuous variables are reported as the mean ± standard deviation (SD) or range. Student's t-test was used to compare the means of continuous variables that had a normal distribution, and the Wilcoxon signed-rank test was used to compare the means of continuous variables that did not have a normal distribution. The chi-squared test or Fisher’s exact test was used to compare the proportions of categorical variables (e.g., sex) between groups. The alpha level was set to 0.05. The significance level was set to p < 0.05 for two-sided tests.

Survival curves were estimated using the Kaplan–Meier method, and life expectancy between the two groups was assessed using the log-rank test. The multivariate Cox proportional hazards model was used to estimate the adjusted hazard ratio (HR) with the 95% confidence interval. Multivariate regression analysis was performed using age, sex, smoking history, histology, presence of concomitant platinum use, presence of TKI use, recurrence, performance status, and COPD in the COPD-non-COPD group comparison. Multivariate regression analysis of COPD patients was performed using age, sex, platinum use (yes or no), TKI use (yes or no), performance status, COPD severity, and COPD treatment (yes or no).

All statistical analyses were performed using the statistical software JMP Pro 14.0 for Windows (SAS Institute Inc, Tokyo, Japan, www.jmp.com). Kaplan–Mayer curves were visualized by GraphPad Prism 9.2 for Windows (GraphPad Software, San Diego, CA, www.graphpad.com).

## Results

### Patient characteristics with advanced NSCLC

Detailed demographic clinical information is shown in Table 1. Of the 550 patients who underwent chemotherapy for recurrence after curative treatment and locally advanced or metastatic NSCLC, 347 patients (63.1%) who underwent pulmonary function tests were analyzed. Of them, 103 patients (18.9%) had COPD (COPD group), 219 (39.8%) did not (non-COPD group), and 25 were excluded due to diagnosis of asthma and radiation pneumonitis. As we expected, sex, smoking status, tyrosine kinase inhibitor (TKI) treatment, histology and PS were significantly different between the COPD group and the non-COPD group.

**Table 1:** Clinical characteristics of patients with advanced NSCLC.

Characteristic	Non-COPD n = 219	COPD n = 103	*P* value
Age (years)	67.0 ± 10.2^a^	68.6 ± 7.77^a^	0.16^b^
Sex, male (%)	118 (53.9)	94 (91.3)	< *0.001*^c^
**Smoking status**			< *0.001*^c^
Nonsmoker	112 (51.1)	0	
Former smoker	63 (28.8)	44 (42.7)	
Current smoker	44 (20.1)	59 (57.2)	
**Chemotherapy**			
Platinum doublet	138 (63.0)	61 (59.2)	0.54
TKI	131 (59.8)	36 (35.0)	< *0.001*
**Histology**			*0.0001*^c^
Squamous	26 (11.9)	29 (28.2)	
Adeno	177 (80.8)	59 (57.3)	
NSCLC	11 (5.0)	12 (11.7)	
Other	5 (2.3)	3 (2.9)	
**NSCLC stage**			0.90
4	151 (69.0)	70 (68.0)	
Recurrence	68 (31.1)	33 (32.0)	
Surgery/radiotherapy/chemoradiotherapy	40/6/26	14/5/15	
**Performance status**			*0.0136*^c^
0	119 (54.3)	44 (42.7)	
1	86 (39.3)	47 (45.6)	
2	9 (4.1)	12 (11.7)	
3	5 (2.3)	0 (0)	

Continuous variables are presented as the mean, and categorical variables are presented as the number (%). Comparisons were made by means of chi-squared tests unless otherwise indicated.

Significant values are in [italics].

*TKI* Tyrosine kinase inhibitors, *NSCLC* Non-small-cell lung cancer.

^a^Mean ± SD.

^b^Student’s t-test.

^c^Fisher’s exact test.

Among the COPD group, 37 patients in the COPD group had received pharmacological treatment for COPD, mainly using LABDs. Thirty-three (89.1%) patients were treated with tiotropium, a long-acting muscarinic antagonist (LAMA). Other patients were treated with a long-acting beta agonist (LABA) alone or in combination with an inhaled corticosteroid (ICS), and 13 (36.1%) patients were treated with all three compounds (Table 2)^32^.

**Table 2:** Univariate and multivariate analyses of the association between various clinical characteristics and OS.

Variable	Univariate		Multivariate	
	HR (95% CI)	*P* value	HRadj (95% CI)	*P* value
Age, < 75 years	0.73 (0.56–0.96)	*0.021*	0.89 (0.64–1.24)	0.48
Sex, male	1.76 (1.36–2.30)	< *0.001*	1.36 (0.89–2.06)	0.15
Smoking status, ≥ 10 pack-years	1.97 (1.52–2.58)	< *0.001*	1.17 (0.74–1.86)	0.51
Histology, squamous	1.88 (1.39–2.56)	< *0.001*	1.19 (0.84–1.68)	0.32
**Chemotherapy**				
Platinum doublet	0.79 (0.62–1.01)	0.060	0.83 (0.61–1.14)	0.26
TKI	0.52 (0.41–0.67)	< *0.001*	0.66 (0.49–0.88)	*0.0047*
Recurrence	0.62 (0.47–0.80)	*0.0004*	0.62 (0.47–0.83)	*0.0011*
Performance status, ≥ 2	3.92 (2.53–5.84)	< *0.001*	3.36 (2.13–5.31)	< *0.001*
COPD	1.62 (1.25–2.09)	*0.0002*	1.06 (0.78–1.43)	0.72

Comparisons were made by means of chi-squared tests unless otherwise indicated.

Significant values are in [italics].

*TKI* Tyrosine kinase inhibitors.

### COPD and OS in patients with advanced NSCLC

**Figure 1:**
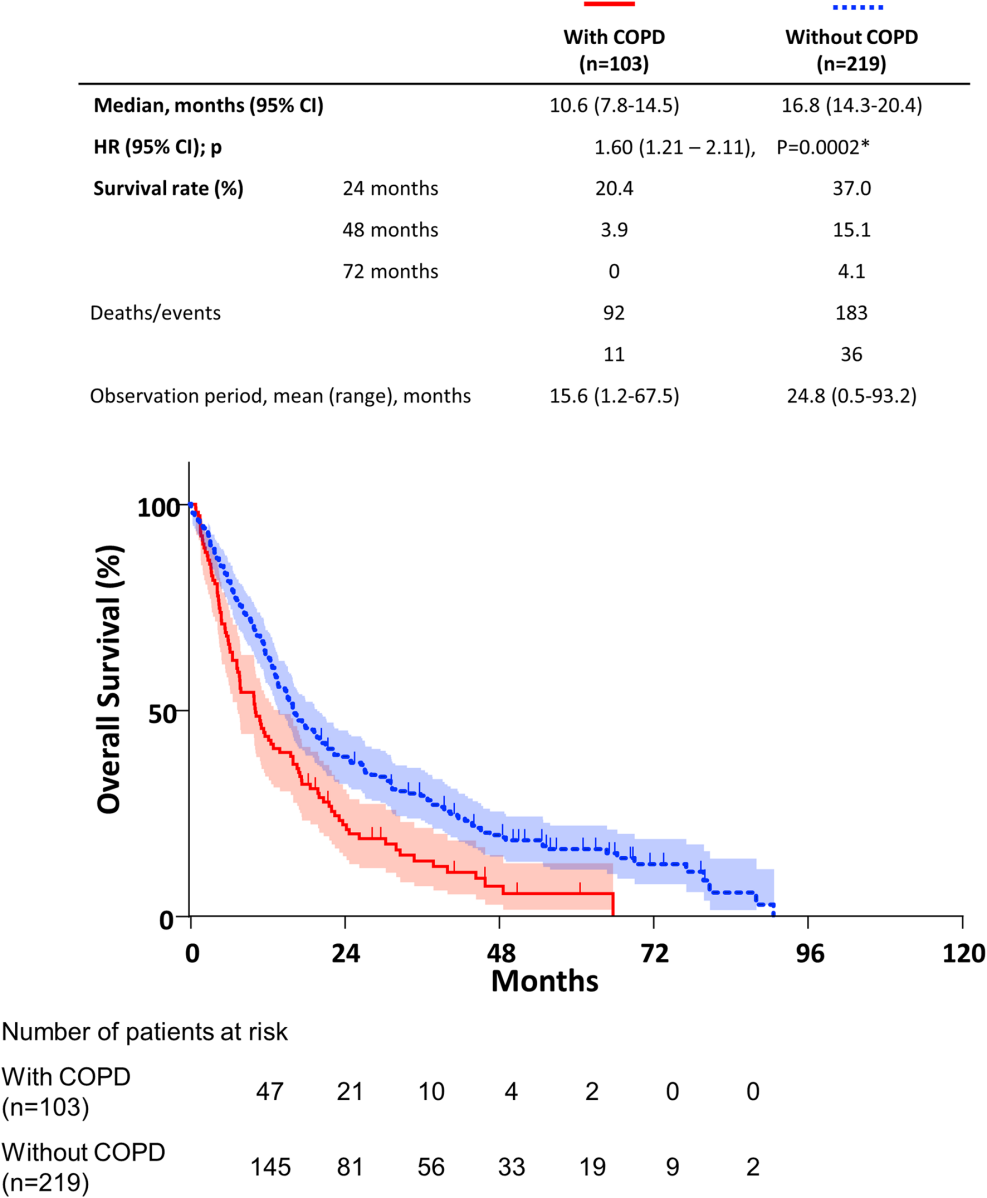
Kaplan–Meier curve of OS stratified by COPD. Coexisting COPD was associated with a significantly shorter OS in advanced NSCLC patients (*P* = 0.0002*, log-rank test). *CI* confidence interval, *HR* hazard ratio.

Kaplan–Meier curves and log-rank tests showed that COPD was associated with significantly shorter OS in advanced NSCLC (Fig. 1). The median OS in the COPD group (10.6 months) was lower than that in the non-COPD group (16.8 months).

In the univariate Cox proportional hazard model, after adjusting for clinicopathologic variables, sex (male), smoking status (≥ 10 pack-years), histology (squamous carcinoma), non-TKI treatment and better PS were significantly associated with worse OS in patients with advanced NSCLC (Table 2).

In the multivariate Cox proportional hazard model, after adjusting for clinicopathologic variables, TKI treatment, recurrence after curative treatment and better PS were significantly associated with better OS in patients with advanced NSCLC (Table 2). However, the adjusted hazard ratio (HRadj) for COPD patients compared with non-COPD patients was 1.06 (95% confidence interval (CI) 0.78–1.43).

### Patient characteristics in the COPD group

Detailed demographic clinical information of the COPD group is shown in Table 3. Classification of airflow limitation severity in COPD (GOLD) and recurrence were significantly different between the patients who received COPD treatment and those who did not. The proportion of patients with severe COPD was 37.8% in the COPD treatment group and 6.1% in the group without COPD treatment. The proportion of patients with recurrence in the patients with and without COPD treatment was 46% and 24.2%, respectively.

**Table 3:** Clinical characteristics in COPD group.

Characteristic	With COPD treatment n = 37	Without COPD treatment n = 66	*P* value
Age (years)	68.0 ± 7.8^a^	69.0 ± 7.8^a^	0.84^b^
Sex, male	33 (89.2)	61 (92.4)	0.72^c^
**Smoking status**			0.94
Nonsmoker	0	0	
Former smoker	16 (43.2)	28 (42.4)	
Current smoker	21 (56.8)	38 (57.6)	
**GOLD**			*0.0002*^c^
1	4 (10.8)	20 (30.3)	
2	19 (51.4)	42 (63.6)	
3	11 (29.7)	4 (6.1)	
4	3 (8.1)	0 (0)	
**COPD treatment**			
LAMA	32 (86.5)	–	
ICS/LABA	20 (54.1)	–	
Triple therapy	13 (35.1)	–	
**Comorbidities**			
Interstitial pneumonitis	3 (8.1)	1 (1.5)	0.13^c^
History of cardiovascular events	4 (10.8)	11(16.7)	0.56^c^
**Chemotherapy**			
Platinum doublet	21 (56.8)	40 (60.6)	0.70
TKI	9 (24.3)	27 (40.9)	0.13^c^
Number of regimens (range)	3.16 (1–13)	2.39 (1–11)	0.45^b^
**Histology**			0.63^c^
Squamous	10 (27.0)	19 (28.8)	
Adeno	22 (59.5)	37 (56.1)	
NSCLC	3 (8.1)	9 (13.6)	
Other	2 (5.4)	1 (1.5)	
**NSCLC stage**			*0.0248*
4	20 (54.1)	50 (75.8)	
Recurrence	17 (46.0)	16 (24.2)	
Surgery/radiotherapy/chemoradiotherapy	6/3/9	8/2/6	
**Performance status**			0.84^c^
0	16 (43.2)	28 (42.4)	
1	16 (43.2)	31 (47.0)	
2	5 (13.5)	7 (10.6)	
3	0 (0)	0 (0)	
4	0 (0)	0 (0)	

Continuous variables are presented as the mean, and categorical variables are presented as the number (%). Comparisons were made by means of chi-squared tests unless otherwise indicated.

Significant values are in [italics].

*GOLD* The Global Initiative for Chronic Obstructive Lung Disease, *LAMA* Long-acting muscarinic antagonist, *ICS* Inhaled corticosteroid, *LABA* Long-acting beta agonist, *TKI* Tyrosine kinase inhibitors, *NSCLC* Non-small-cell lung cancer.

^a^Mean ± SD.

^b^Wilcoxon signed-rank test.

^c^Fisher’s exact test.

### COPD treatment and OS

**Figure 2:**
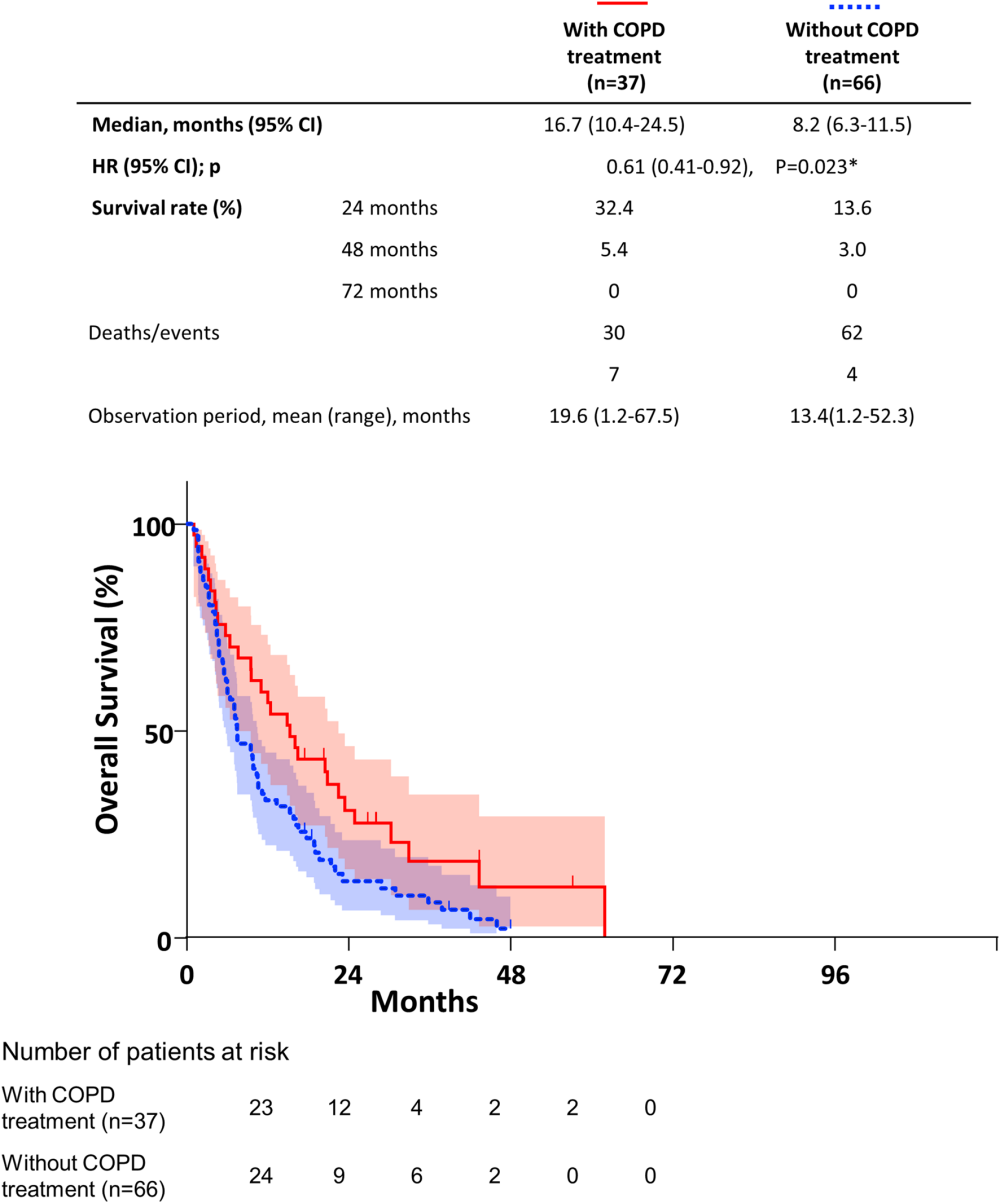
Kaplan–Meier curve of OS stratified by COPD treatment. COPD treatment was associated with a significantly longer OS in advanced NSCLC patients (*P* = 0.023*, log-rank test). *CI* confidence interval, *HR* hazard ratio.

Kaplan–Meier curves and log-rank tests showed that COPD treatment was associated with significantly longer OS in advanced NSCLC (Fig. 2). The median OS in patients without COPD treatment (8.2 months) was lower than that in patients with COPD treatment (16.8 months). In the univariate Cox proportional hazard model, recurrence, better PS and COPD treatment were significantly associated with better OS in the COPD group (Table 4). In the multivariate Cox proportional hazard model, recurrence, mild COPD stage, better PS, and COPD treatment were significantly associated with better OS in patients with advanced NSCLC, and the HRadj for COPD treatment was 0.52 (95% CI 0.31–0.87).

**Table 4:** Univariate and multivariate analyses of the association between various clinical characteristics and OS in the COPD group.

Variable	Univariate		Multivariate	
	HR (95% CI)	*P* value	HRadj (95% CI)	*P* value
Age, < 75 years	0.89 (0.56–1.45)	0.63	1.16 (0.68–1.97)	0.59
Sex, male	0.94 (0.48–2.11)	0.86	1.14 (0.52–2.50)	0.75
Recurrence	0.49 (0.30–0.78)	*0.0037*	0.45 (0.27–0.76)	*0.0017*
**Chemotherapy**				
Platinum doublet	0.79 (0.52–1.20)	0.26	0.76 (0.48–1.23)	0.27
TKI	0.94 (0.60–1.45)	0.79	0.76 (0.47–1.23)	0.25
%FEV1 < 80%	1.48 (0.92–2.47)	0.12	2.63 (1.53–4.70)	*0.0007*
Performance status, ≥ 2	2.96 (1.51–5.31)	*0.0006*	2.79 (1.40–5.57)	*0.0037*
COPD treatment, yes	0.60 (0.38–0.93)	*0.0243*	0.52 (0.31–0.87)	*0.0134*

Comparisons were made by means of chi-squared tests unless otherwise indicated.

Significant values are in [italics].

*TKI* Tyrosine kinase inhibitors, *FEV1* forced expiratory volume in 1 s.

## Discussion

Here, we found that coexisting COPD might be associated with a worse survival outcome in patients with advanced NSCLC in our observational retrospective cohort study. Interestingly, among patients with coexisting COPD, more than half of the patients did not receive pharmacological treatment for COPD, and these patients had a substantially worse prognosis than patients with treatment for COPD. Our results indicated that even in patients with advanced NSCLC, coexisting COPD must be treated accordingly. To the best of our knowledge, this is the first study to identify the survival impact of pharmacotherapy for coexisting COPD in patients with advanced NSCLC.

COPD is often a comorbid condition in patients with lung cancer, but its effect on survival is not well understood. In the present study, almost half of the patients who had a smoking history and spirometry had COPD (coexisting COPD). These patients tended to be male, infrequently treated without TKIs, infrequently diagnosed without squamous carcinoma and have a poorer PS than patients without COPD. These factors may be associated with a poor prognosis. Although univariate Cox regression analysis revealed a negative impact of coexisting COPD for patients with advanced lung cancer, multivariate regression analysis showed that coexisting COPD did not have a significant impact on OS. These results are somehow inconsistent with those of previous studies. A meta-analysis including 21 studies (60,764 participants and 11,270 cases) revealed that COPD had a significant impact on OS in lung cancer patients^33^. However, several reports show that COPD does not have a significant deleterious impact on OS^34,35^. A previous meta-analysis also showed that the impact of coexisting COPD may be smaller in patients with advanced cancer than in patients with early-stage cancer^33^.

Notably, the median OS of patients with treatment for coexisting COPD was significantly prolonged relative to that of the patients without treatment (16.7 vs. 8.2 months, Fig. 1) and was equivalent to that in the non-COPD groups (16.8 months, Supplemental Fig. S1, Additional File 1). Since all of the patients were treated according to guidelines for COPD in a previous study^34^, our results are quite consistent with those of previous studies.

On the other hand, a lack of treatment for coexisting COPD is an important issue. In the present study, 64% of the patients with coexisting COPD did not receive treatment for COPD, including LABDs. Furthermore, the patients without COPD treatment had a lower proportion of severe to very severe COPD than those with COPD treatment. The proportions of patients with mild to moderate COPD and severe to very severe COPD in the patients with treatment were 62.2% and 37.8%, whereas in the patients without treatment, they were 93.9% and 6.1%, respectively. However, mild COPD patients may not complain much compared to severe COPD patients and may not request treatment for their symptoms. As a result, the patients without treatment showed a worse survival outcome despite the milder severity of COPD. These results suggest that we must consider pharmacological treatment for coexisting COPD to improve OS in patients with advanced NSCLC even in the mild stage of COPD.

Regarding pharmacological treatment for COPD, three major classes are currently available, including LAMAs, LABAs, and ICSs^32^. Treatment with these drugs (mono- or combination therapy) was associated with improvements in lung function, exercise capacity, quality of life, the rate of exacerbations, and prognosis^21–25^. In this study, 89.1% of patients with COPD treatment were treated with tiotropium (LAMA). During our enrolment period of April 2007 to March 2014, tiotropium and combined fluticasone/salmeterol were the most popular medications. Due to the small sample size and because 13 patients used combined medications (such as “triple therapy”), performing an additional analysis to compare the effects of two medications in the present study was difficult; however, pharmacological treatment for COPD might contribute to preventing a PS decline in advanced NSCLC patients with COPD.

The mechanisms underlying the positive impact on the prognosis of advanced NSCLC patients with coexisting COPD are still unclear, and the biological mechanisms related to the anti-oncogenesis or anti-inflammatory effects are uncertain^36,37^, although a possible mechanism may be prevention of a functional status decline due to breathlessness, resulting in an increased number of chemotherapy regimens. Unfortunately, no significant difference was found in the number of chemotherapy regimens between the two groups, whereas according to the effect of palliative care, appropriate treatment and care for breathlessness may have a positive impact on OS^38^. Patients with lung cancer and coexisting COPD who received care from a pulmonologist were significantly more likely to undergo surgery and experience improved survival^39^. Pulmonologists might improve symptom management and decrease respiratory complications by preventing functional status declines.

A limitation of this study is the small sample size from a single center. Therefore, unexpected confounding biases and the influence of data deficiency cannot be excluded. Since this study was retrospective cohort, the choice of whether or not to perform spirometry was not random, and confounding bias could not be excluded. As shown in Table S2, patients who were excluded from the study due to no spirometry had mostly stage 4 disease and a relatively poor PS (Table S2, Additional File 1). In fact, these patients may not have benefited from COPD treatment if they had COPD. However, if they had undergone spirometry before the worsening of their condition, COPD treatments may have helped to improve their condition to some extent. We think that physicians should be encouraged to perform spirometry to detect COPD in patients with lung cancer, and if they have COPD, COPD treatments should be considered. Moreover, there was the possibility that patients might be treated for COPD due to the physician's expectations regarding the long-term prognosis or expectation of good organ function. In most cases of patients who had COPD in their medical records but did not receive treatment, there was no reason noted in the medical record, even though some of the patients clearly had COPD (Table S3, Additional File 1). However, due to the supportive results in the univariate and multivariate analyses, we believe that potential biases were adequately considered and may not have had a considerable impact on the present analysis. To overcome this limitation, we are currently undergoing a multi-institutional joint study to confirm the efficacy of COPD treatment in NSCLC patients with COPD. Another limitation is that patients who received immunotherapy were excluded from the present analysis. Because ICIs have an extensive impact on survival in advanced NSCLC patients and have already changed the basic chemotherapy strategies for NSCLC, we had to eliminate these effects in the present study. Moreover, regarding this issue, we performed an additional validation analysis on patients with advanced NSCLC who were treated with ICIs after 2016 (“Supplemental information”, Additional File 1), and we were able to confirm similar positive impacts of treatment for coexisting COPD (by log-rank test, *P* = 0.036, Supplemental Table S1, Figs. S3, S4, Additional File 1). Since strategies involving ICIs may significantly prolong the expected survival of advanced NSCLC patients, treatment for coexisting chronic diseases, such as COPD should be appropriately considered.

The current guidelines for lung cancer treatment do not describe how to treat lung cancer with COPD and are mostly dedicated to describing recent advances in genomic-targeted therapy for advanced NSCLC^40^. With regard to ICIs, the anti-PD-1/PD-L1 antibody had more curative properties in current or former smokers^41^. Therefore, considering management of coexisting COPD in patients with advanced NSCLC should become more important. Symptoms of COPD, such as dyspnea on exertion, sputum, and cough, may be masked by symptoms of advanced lung cancer. Our findings have clinical implications for advanced NSCLC, especially in an era when “living with cancer and comorbidities” is required.

## Conclusion

We found that untreated coexisting COPD may have a considerable impact on the prognosis of patients with advanced NSCLC. Pharmacological treatment for coexisting COPD, might have the potential to improve the prognosis of patients with advanced NSCLC with or without treatment with ICIs.

## Supplementary Information

Supplementary Information.

## Data Availability

The datasets used and analyzed during the current study are available from the corresponding author upon reasonable request.

## Abbreviations

NSCLC: Non-small-cell lung cancer
BASCs: Bronchoalveolar stem cells
COPD: Chronic obstructive pulmonary disease
OS: Overall survival
PS: Performance status
HRadj: Adjusted hazard ratio
CI: Confidence interval
BMI: Body mass index
FEV1: Forced expiratory volume in 1 s
FVC: Forced vital capacity
GOLD: Global Initiative for Chronic Obstructive Lung Disease
LABDs: Long-acting bronchodilators
LAMA: Long-acting muscarinic antagonist
ICS: Inhaled corticosteroid
LABA: Long-acting beta agonist
TKI: Tyrosine kinase inhibitors
ICIs: Immune checkpoint inhibitors
