# Author Correction: Survival impact of treatment for chronic obstructive pulmonary disease in patients with advanced non-small-cell lung cancer

**DOI:** 10.1038/s41598-023-29102-0

**Published:** 2023-02-06

**Authors:** Hitomi Ajimizu, Hiroaki Ozasa, Susumu Sato, Tomoko Funazo, Yuichi Sakamori, Takashi Nomizo, Kiyomitsu Kuninaga, Tatsuya Ogimoto, Kazutaka Hosoya, Masatoshi Yamazoe, Takahiro Tsuji, Hironori Yoshida, Ryo Itotani, Kentaro Ueno, Young Hak Kim, Shigeo Muro, Toyohiro Hirai

**Affiliations:** 1 Department of Respiratory Medicine, Kyoto University Graduate School of Medicine, 54 Kawahara-cho, Shogoin, Sakyo-ku, Kyoto, 606-8507 Japan; 2 Department of Respiratory Medicine, Nara Medical University, 840 Shijo-cho, Kashihara, Nara 634-8522 Japan; 3 Department of Biomedical Statistics and Bioinformatics, Graduate School of Medicine, Kyoto University, 54 Kawahara-cho, Shogoin, Sakyo-ku, Kyoto, 606-8507 Japan

Correction to: *Scientific Reports* 10.1038/s41598-021-03139-5, published online 08 December 2021

The original version of this Article contained errors in the Results and Discussion sections, Tables 2 and 4 and in the Supplementary Information.

In the Results section, under the subheading ‘COPD and OS in patients with advanced NSCLC’,

“In the multivariate Cox proportional hazard model, TKI treatment, recurrence after curative treatment and better PS were significantly associated with better OS in patients with advanced NSCLC (Table 2).”

now reads:

“In the multivariate Cox proportional hazard model, after adjusting for clinicopathologic variables, TKI treatment, recurrence after curative treatment and better PS were significantly associated with better OS in patients with advanced NSCLC (Table 2).”

In addition, in the Results section, under the subheading ‘COPD treatment and OS’,

“The median OS in patients with COPD treatment (8.2 months) was lower than that in patients without COPD treatment (16.8 months).”

now reads:

“The median OS in patients without COPD treatment (8.2 months) was lower than that in patients with COPD treatment (16.8 months).”

In addition, in the Discussion section,

“with TKIs, infrequently diagnosed with squamous carcinoma and have a poorer PS than patients”

now reads:

“without TKIs, infrequently diagnosed without squamous carcinoma and have a poorer PS than patients”

Furthermore, the original version of this Article contained errors in Table 2 and Table 4, where “HR (95% CI)” was incorrectly given as “HRadj (95% CI)” under “Univariate”. The correct heading is as below:

Incorrect:

**Table:** 

Variable	Univariate	
	HRadj (95% CI)	*P* value

Correct:

**Table:** 

Variable	Univariate	
	HR (95% CI)	*P* value

The Supplementary Information file published with this Article contained an error in the Supplementary Figure 4, where under the Number of patients at risk “Without COPD treatment (n = 28)” and “With COPD treatment (n = 14)” was interchanged.

This error has now been corrected in the Supplementary Information file that accompanies the original Article.

The original Article has been corrected.

## Supplementary Information

Supplementary Information.

