# Supplementary material for: Survival impact of treatment for chronic obstructive pulmonary disease in patients with advanced non-small-cell lung cancer
Source: Sci Rep. 2021 Dec 8;11:23677. doi: 10.1038/s41598-021-03139-5 (PMC8654854; doi:10.1038/s41598-021-03139-5)
Supplement: Supplementary file 1 — Supplementary Information. [file 41598_2021_3139_MOESM1_ESM.docx]

**SUPPLEMENTARY INFORMATION**

**TITLE:** Survival Impact of Treatment for Chronic Obstructive Pulmonary Disease in Patients with Advanced Non-small-cell Lung Cancer

**Authors’ full names:**

Hitomi Ajimizu^1)^, Hiroaki Ozasa^1)^, Susumu Sato^1)^, Tomoko Funazo^1)^, Yuichi Sakamori^1)^, Takashi Nomizo^1)^, Kiyomitsu Kuninaga^1)^, Tatsuya Ogimoto^1)^, Kazutaka Hosoya^1)^, Masatoshi Yamazoe^1)^, Takahiro Tsuji^1)^, Hironori Yoshida^1)^, Ryo Itotani^1)^, Kentaro Ueno^3)^, Young Hak Kim^1)^, Shigeo Muro^2)^, Toyohiro Hirai^1)^

**Authors’ affiliations:**

^1^Department of Respiratory Medicine, Kyoto University Graduate School of Medicine, 54 Kawahara-cho, Shogoin, Sakyo-ku, Kyoto 606-8507, Japan

^2^Department of Respiratory Medicine, Nara Medical University, 840 Shijo-cho, Kashihara, Nara, 634-8522, Japan

^3^Department of Biomedical Statistics and Bioinformatics, Kyoto University, Graduate School of Medicine, 54 Kawahara-cho, Shogoin, Sakyo-ku, Kyoto 606-8507, Japan

Appendix S1- Patients for an additional validation study.

Appendix S2- Results of an additional validation study.

Figure S1. Study flow chart.

Figure S2. Kaplan-Meier curve of OS for the non-COPD group and COPD patients who received COPD treatment (with COPD treatment).

Figure S3. Study flow chart of validation study.

Figure S4. Kaplan-Meier curve of OS of patients with ICIs stratified by COPD treatment.

Table S1. Clinical characteristics of patients in the ICI treatment and coexisting COPD groups.

Table S2. Clinical characteristics of patients excluded from study.

Table S3. Clinical reasons for no pharmacological intervention in the no COPD treatment group.

**Appendix S1- *Patients for the additional validation study***

For an additional validation study, we enrolled patients with lung cancer who received immune checkpoint inhibitors (ICIs) for recurrence after curative treatment and locally advanced or metastatic NSCLC at Kyoto University Hospital from January 2016 to June 2019.

The inclusion and exclusion criteria were the same as those in the original cohort study, except for the use of ICIs. Patients’ vital statuses were confirmed in December 2020 in this additional study.

**Appendix S2- *Results of the additional validation study***

Of the 168 patients who received chemotherapy with ICIs for recurrence after curative treatment, locally advanced or metastatic NSCLC, 115 patients (63.1%) underwent pulmonary function tests. Forty-two patients had COPD, and 14 (33.3%) patients had received pharmacological treatment for COPD.

Kaplan-Meier curves and log-rank tests showed that COPD treatment was associated with significantly longer OS in advanced NSCLC patients who had received ICIs (Figure S5). The median OS of patients with COPD treatment could not be defined, whereas the median OS of patients without COPD treatment was 19.4 months. The log-rank test showed significant differences in overall survival (OS) between patients with and without COPD treatment (P=0.0355).

**Figure S1.** **Study flow chart.**

**
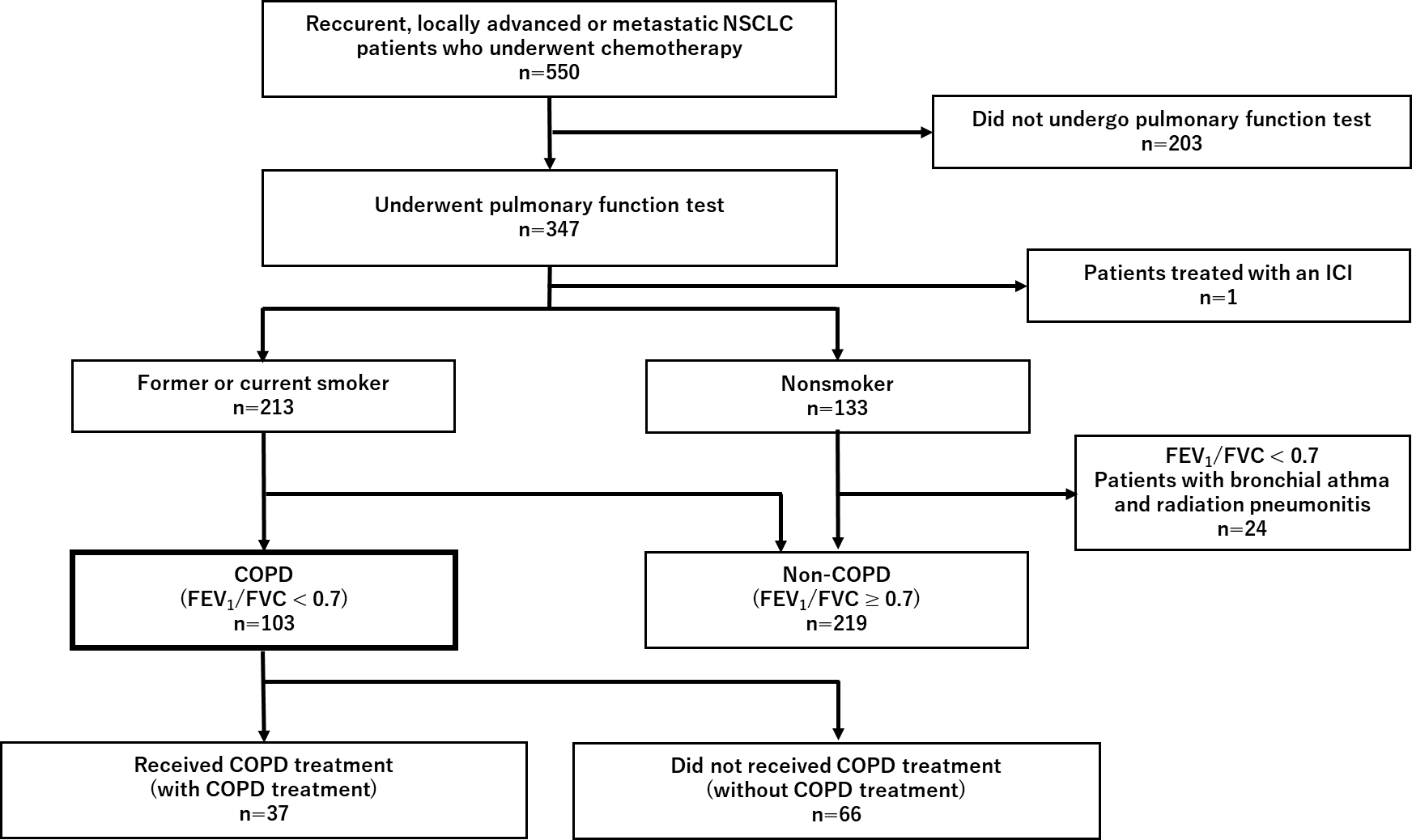
**

**Figure S2***.* **Kaplan-Meier curve of OS of the non-COPD group (without COPD) and COPD patients who received COPD treatment (with COPD treatment).**

The overall survival of COPD patients who received treatment was not significantly different from that of patients without COPD (*P*=0.85*, log-rank test). CI, confidence interval; HR, hazard ratio.

**
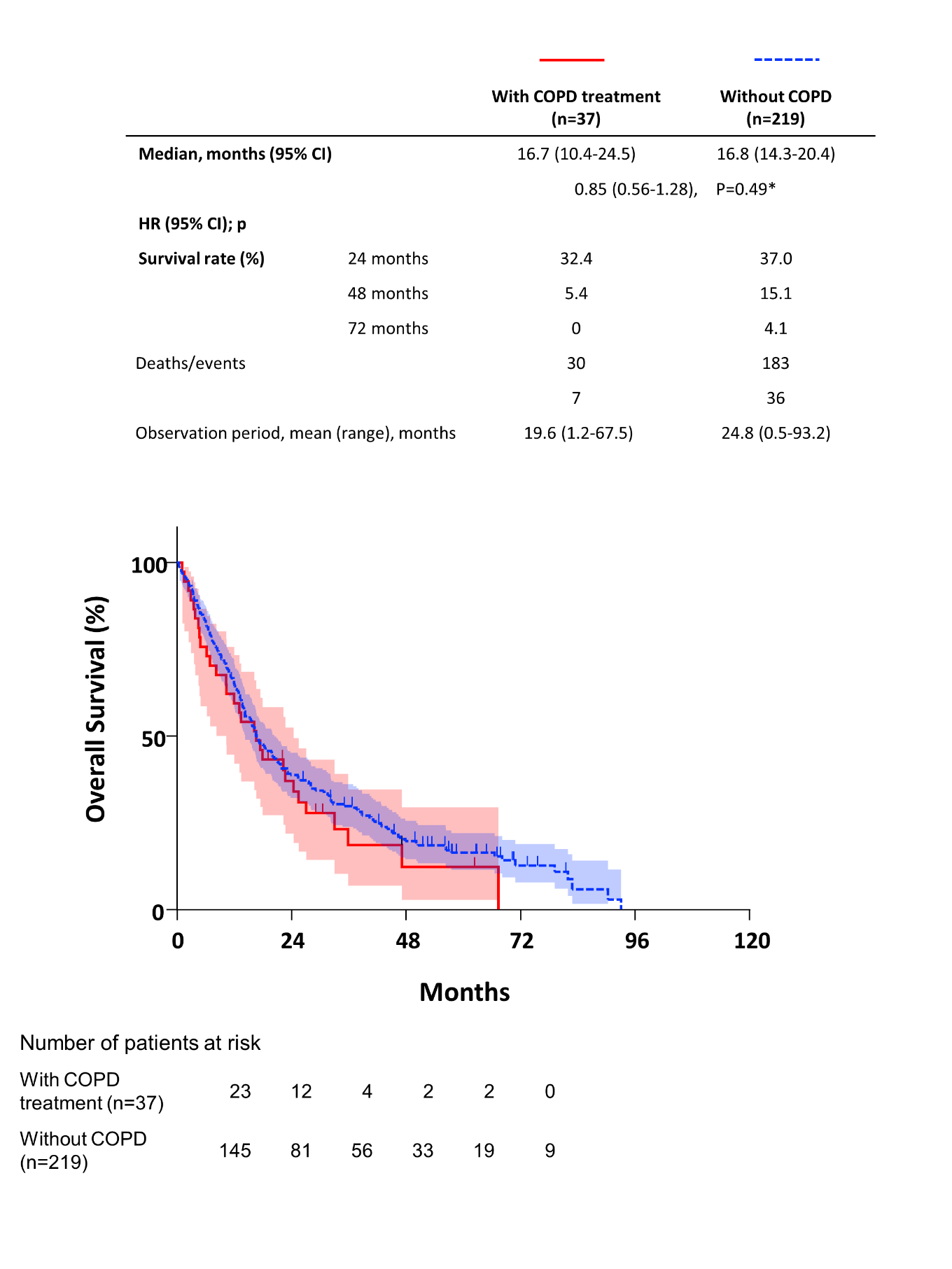
**

**Figure S3***.* **Study flow chart of validation study.**

**
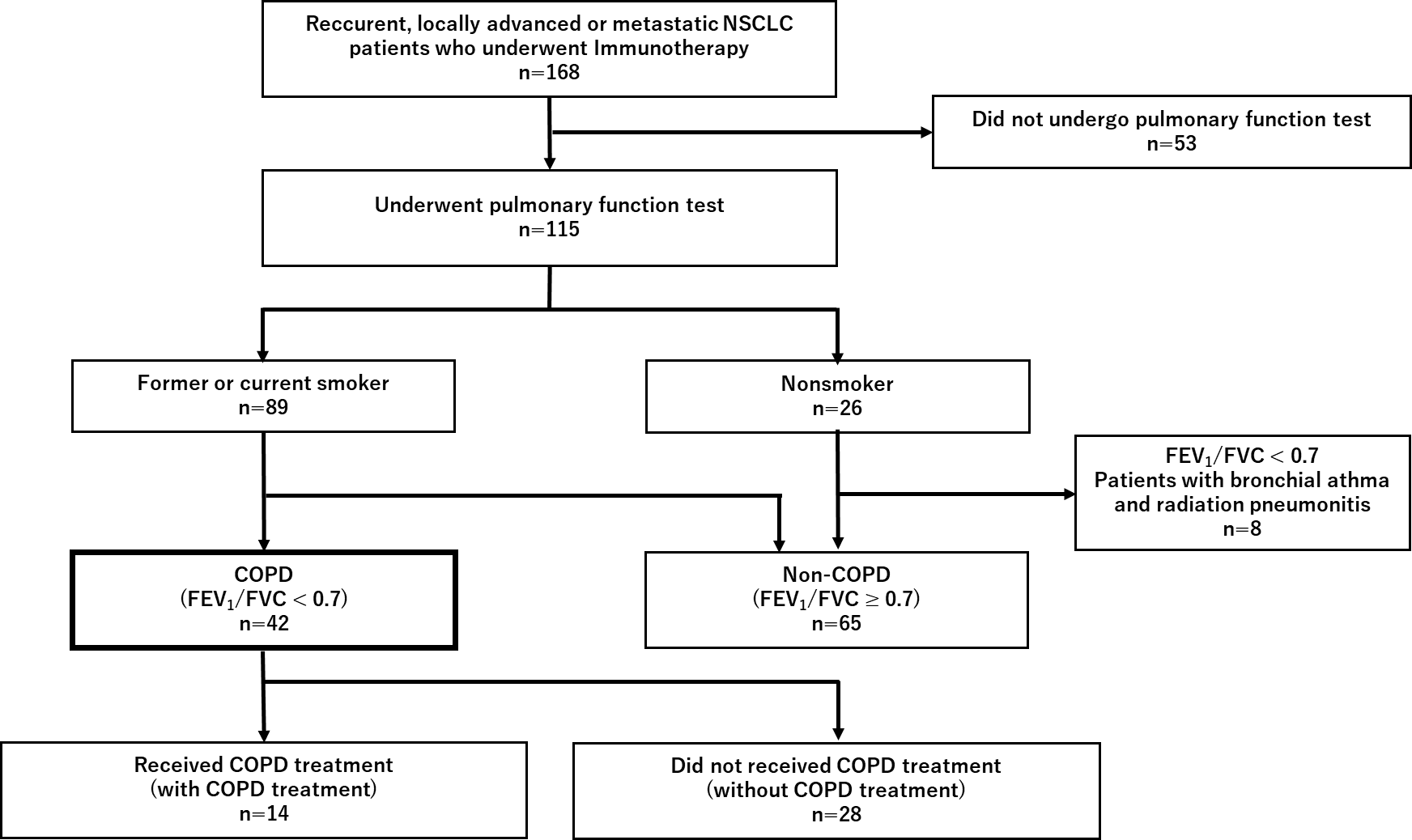
**

**Figure S4. Kaplan-Meier curve of OS of patients with ICIs stratified by COPD treatment.**

COPD treatment was associated with a significantly longer OS in advanced NSCLC patients (*P*=0.036*, log-rank test). CI, confidence interval; NR, not reached; HR, hazard ratio.


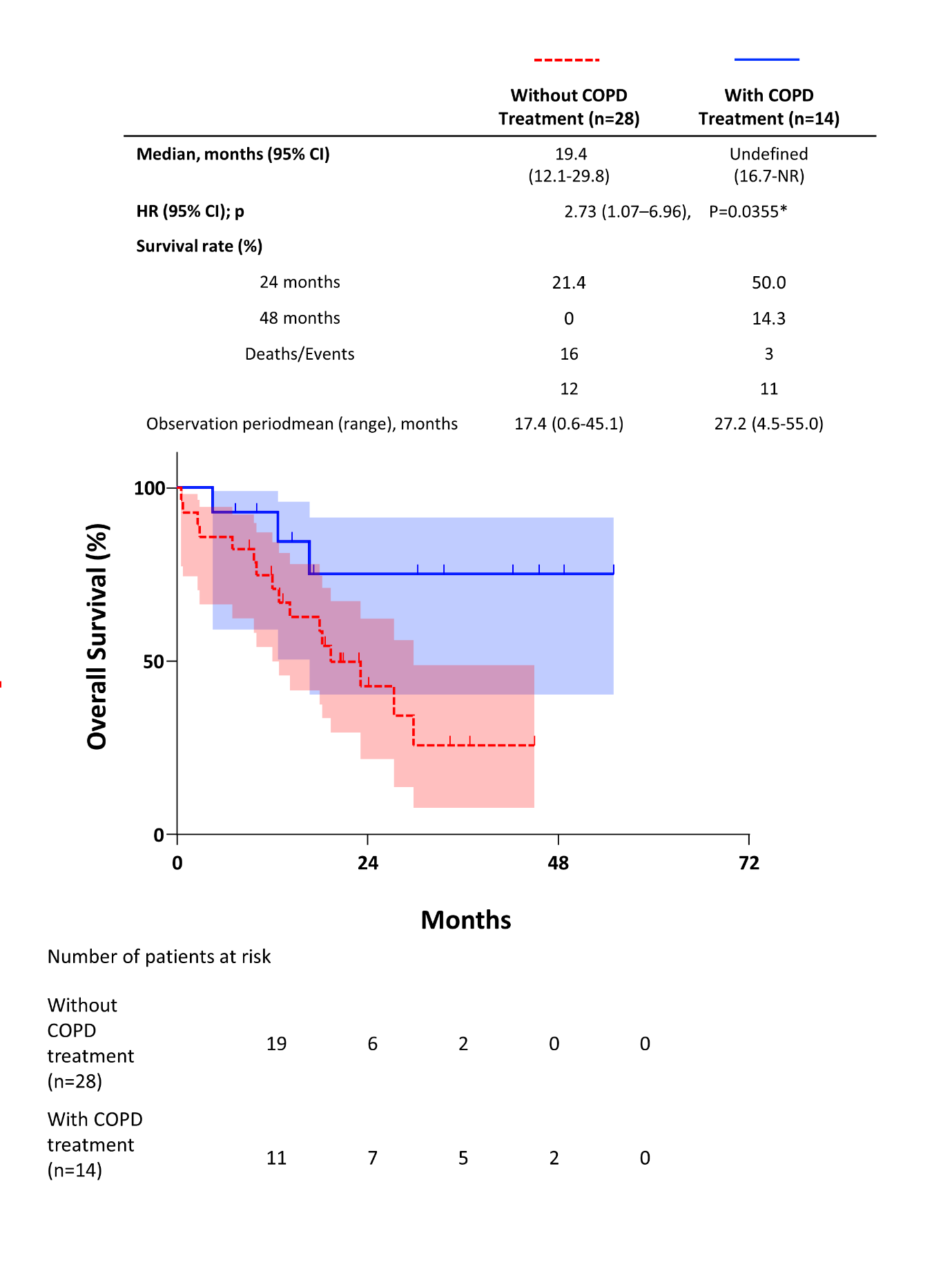


**Table S1. Clinical characteristics of patients in the ICI treatment and coexisting COPD groups.**

| **Characteristic** | **With**  **COPD Treatment**  **n=14** | **Without**  **COPD treatment**  **n=28** | ***P* value** | |
| --- | --- | --- | --- | --- |
| **Age**, years | 73.7±4.5 ^a^ | 72.3±7.4 ^a^ | 0.56 ^c^ | |
| **Sex**, male (%) | 12 (85.7) | 23 (82.1) | 1.00 ^d^ | |
| **Smoking status,**  Nonsmoker  Former smoker  Current smoker |  |  | 1.00 ^d^ | |
|  | 0 (0) | 0 (0) |  | |
|  | 8 (57.1) | 16 (57.1) |  | |
|  | 6 (42.9) | 12 (42.9) |  | |
| **GOLD** |  |  | 0.65 ^d^ | |
| 1 | 4 (28.6) | 10 (35.7) |  | |
| 2 | 10 (71.4) | 16 (57.1) |  | |
| 3 | 0 (0) | 2 (7.1) |  | |
| **COPD treatment** |  |  |  | |
| LAMA | 12 (85.7) |  |  | |
| LABA | 9 (64.3) |  |  | |
| ICS | 3 (21.4) |  |  | |
| **Chemotherapy** |  |  |  | |
| Platinum doublet | 11 (78.6) | 20 (71.4) | 0.72 ^d^ | |
| TKI | 0 (0) | 1 (3.6) | 1.00 ^d^ | |
| Number of regimens  (Mean) | 2.21 | 2.64 | 0.51 ^c^ | |
| **Comorbidities** |  |  |  | |
| Interstitial pneumonitis | 2 (7.1) | 0 (0) | 0.55 ^d^ | |
| History of  cardiovascular events | 5 (17.9) | 2 (14.3) | 1.0 ^d^ | |
| **Histology** |  |  | *0.0234* ^d^ | |
| Squamous | 2 (14.3) | 11 (39.3) |  | |
| Adeno | 11 (78.6) | 12 (42.9) |  | |
| NSCLC | 0 (0) | 5 (17.9) |  | |
| Other | 1 (7.1) | 0 (0) |  | |
| PD-L1 expression, ≥50% | 3 (21.4) | 4 (14.3) | 0.67 ^d^ | |
| **NSCLC stage** |  |  | 0.12 ^d^ | |
| 4 | 5 (35.7) | 17 (60.7) |  | |
| Recurrence | 9 (64.3) | 11 (39.3) |  | |
| Surgery/radiotherapy/  chemoradiotherapy | 6/3/2 | 6/8/0 |  | |
| **Performance status** |  |  | *0.19* ^d^ | |
| 0 | 11 (78.6) | 14 (50.0) |  | |
| 1 | 3 (21.4) | 11 (39.3) |  | |
| 2 | 0 (0) | 3 (10.7) |  | |
| Continuous variables are presented as the mean, and categorical variables are presented as the number (%). Comparisons were made by means of chi-squared tests unless otherwise indicated. a; mean ± SD. b; Student’s t-test. c; Wilcoxon signed-rank test. d; Fisher’s extract test.  GOLD, The Global Initiative for Chronic Obstructive Lung Disease; LAMA, Long-acting muscarinic antagonist; ICS, Inhaled corticosteroid; LABA, Long-acting beta agonist; PD-L1, Programmed cell Death Ligand 1; TKI, Tyrosine kinase inhibitors; NSCLC, Non-small-cell lung cancer. | | | |  |

**Table S2. Clinical characteristics of patients excluded from the study in the main cohort.**

| **Characteristic** | Patients included  **n=322** | Patients excluded  **n=203** | ***P* value** |
| --- | --- | --- | --- |
| Age, years | 67.5±9.53 ^a^ | 66.0±9.73 ^a^ | 0.31 ^b^ |
| Sex, male (%) | 212 (65.8) | 121 (59.6) | *0*.15 |
| Smoking status,  Nonsmoker  Former smoker  Current smoker |  |  | 0.69 |
|  | 112 (34.8) | 69 (34.0) |  |
|  | 107 (33.2) | 63 (30.5) |  |
|  | 103 (32.0) | 72 (35.5) |  |
| Chemotherapy |  |  |  |
| Platinum doublet | 199 (61.8) | 131 (64.5) | 0.53 |
| TKI | 167 (51.9) | 97 (47.8) | 0.36 |
| Histology |  |  | 0.11 ^c^ |
| Squamous | 55 (17.1) | 22 (10.8) |  |
| Adeno | 236 (73.3) | 153 (75.4) |  |
| NSCLC | 23 (7.1) | 23 (11.3) |  |
| Other | 8 (2.5) | 5 (2.5) |  |
| NSCLC stage |  |  | *<0.001* |
| 4 | 221 (68.6) | 175 (86.2) |  |
| Recurrence | 68 (31.1) | 28 (13.8) |  |
| performance status |  |  | *0.008* ^c^ |
| 0 | 163 (50.6) | 93 (45.8) |  |
| 1 | 133 (41.3) | 69 (34.0) |  |
| 2 | 21 (6.5) | 30 (14.8) |  |
| 3 | 5 (1.5) | 11 (5.4) |  |
| Continuous variables are presented as the mean, and categorical variables are presented as the number (%). Comparisons were made by means of chi-squared tests unless otherwise indicated. a; mean ± SD. b; Student’s t-test. c; Fisher’s extract test.  TKI, Tyrosine kinase inhibitors; NSCLC, Non-small-cell lung cancer. | | | |

**Table S3. Clinical reasons for no pharmacological intervention in the group without COPD treatment in the main cohort.**

| Reason |  |
| --- | --- |
| No therapeutic intervention: relatively mild disease and/or lack of subjective symptoms | 10 (15.2%) |
| No intervention: False positive due to pleural effusion or lung cancer | 1 (1.5%) |
| Difficult to intervene in treatment: Decreased ADL, difficulty using inhalants | 1 (1.5%) |
| Difficulty in continuing treatment: Adverse effects from inhalants | 0 (0%) |
| Difficulty in continuing treatment: Rapid deterioration of cancer condition | 0 (0%) |
| Difficulty in continuing treatment: Poor compliance | 0 (0%) |
| No entry in medical record (including ignorance) | 54 (81.8%) |
